# AN ANALYSIS OF PALLIATIVE CARE PROVIDER NURSING HOME CONSULTATIONS FOR PATIENTS WITH COGNITIVE IMPAIRMENT

**DOI:** 10.1093/geroni/igae098.2493

**Published:** 2024-12-31

**Authors:** Matthew Nesvet, Alex Floyd, John Cagle, Hanley Elftmann, Kathleen Unroe

**Affiliations:** Regenstrief Institute (IUCAR), and MDC, Indianapolis, Indiana, United States; Indiana University School of Medicine, Indianapolis, Indiana, United States; University of Maryland Baltimore, Baltimore, Maryland, United States; Indiana University School of Medicine, Indianapolis, Indiana, United States; Indiana University, Indianapolis, Indiana, United States

## Abstract

Nursing homes (NH) are important sites of care for people with serious illnesses in need of end-of-life care. Extant studies have shown NH residents with dementia struggle to receive high quality care. There is some evidence that palliative care (PC) interventions may improve quality of life (QOL) and care. There is little prior description of PC consults conducted in the nursing home setting. We examined notes PC providers wrote after initial consults with 197 residents of 8 nursing homes enrolled in UPLIFT, an NIH-funded clinical trial evaluating implementation of a PC model of care. Residents enrolled in UPLIFT who screened positive for PC needs were referred for consultations. We analyzed initial consult notes to learn about the residents referred and content of the consultative visits, including recommendations made. Mean age of the residents was 75 years. 70% were White, 19% Black/African American, and 52% female. Goals of care were discussed in 83% of the PC consults, the most commonly observed intervention. The most frequent types of recommendations by PC providers were: 1) medication-related (n=207); 2) referrals to other clinical services (n=103); 3) non-pharmacologic symptom relief (n=53); and 4) specific recommendations for NH staff-based activities (n=40). In total, PC providers averaged three recommendations when at least one family member, friend, or guardian was present during the clinical encounter in person (n=127) or by phone (n=38), and two when only the resident participated in the encounter (n=33).

